# Enhanced Solar Photocatalytic Activity of Thermally Stable I:ZnO/Glass Beads for Reduction of Cr(VI) in Tannery Effluent

**DOI:** 10.3389/fchem.2022.805913

**Published:** 2022-03-02

**Authors:** Ambreen Ashar, Ijaz Ahmad Bhatti, Muhammad Mohsin, Maryam Yousaf, Humera Aziz, Adeeba Gul, Tausif Hussain, Zeeshan Ahmad Bhutta

**Affiliations:** ^1^ Department of Chemistry, University of Agriculture Faisalabad (UAF), Faisalabad, Pakistan; ^2^ School of Chemistry and Chemical Engineering, Beijing Institute of Technology, Beijing, China; ^3^ Department of Environmental Science and Engineering, Government College University, Faisalabad, Pakistan; ^4^ Centre for Advance Studies in Physics (CASP), Government College University, Lahore, Pakistan; ^5^ Laboratory of Biochemistry and Immunology, College of Veterinary Medicine, Chungbuk National University, Cheongju, South Korea

**Keywords:** solar photocatalysis, I:ZnO/GB, reduction of Cr(VI), tannery effluent, response surface methodology

## Abstract

Chromium (VI) in tannery effluent is one of the major environmental concerns for the environmentalists due to the hazardous nature of Cr(VI) ions. To reduce Cr(VI) to Cr(III) as an innocuous moiety, pure and I-doped ZnO was grafted over the etched surface of glass beads by successive ionic layer adsorption and reaction (SILAR). Powdered, pure, and I-doped ZnO scrapped from the surface of glass beads was characterized for crystallinity, morphology, and elemental composition by XRD, SEM, TEM, and EDX. The optical properties of both photocatalysts revealed that owing to optimized iodine doping of ZnO, reduction in the bandgap was observed from 3.3 to 2.9 eV. The crystalline nano-bricks of I:ZnO adhered to glass beads were investigated to have remarkable capability to harvest sunlight in comparison to intrinsic ZnO nanodiscs. The thermal stability of I:ZnO was also found to be much improved due to doping of ZnO. The photocatalytic activities of ZnO/GB and I:ZnO/GB were compared by extent of reduction of Cr(VI) under direct natural sunlight (600–650 KWh/m^2^). The disappearance of absorbance peaks associated with Cr(VI) after treatment with I:ZnO/GB confirmed higher photocatalytic activity of I:ZnO/GB. The reaction parameters of solar photocatalytic reduction, i.e., initial pH (5–9), initial concentration of Cr(VI) (10–50 ppm), and solar irradiation time (1–5 h) were optimized using response surface methodology. The solar photocatalytic reduction of Cr(VI) to Cr(III) present in real tannery effluent was examined to be 87 and 98%, respectively, by employing ZnO/GB and I:ZnO/GB as solar photocatalysts. The extent of reduction was also confirmed by complexation of Cr(VI) and Cr(III) present in treated and untreated tannery waste with 1, 5-diphenylcarbazide. The results of AAS and UV/vis spectroscopy for the decrease in concentration of Cr also supported the evidence of higher efficiency of I:ZnO/GB for reduction of Cr(VI) in tannery effluent. Reusability of the fabricated photocatalyst was assessed for eight cycles, and magnificent extent of reduction of Cr(VI) indicated its high efficiency. Conclusively, I:ZnO/GB is a potential and cost-effective candidate for Cr(VI) reduction in tannery effluent under natural sunlight.

**Graphical Abstract:**
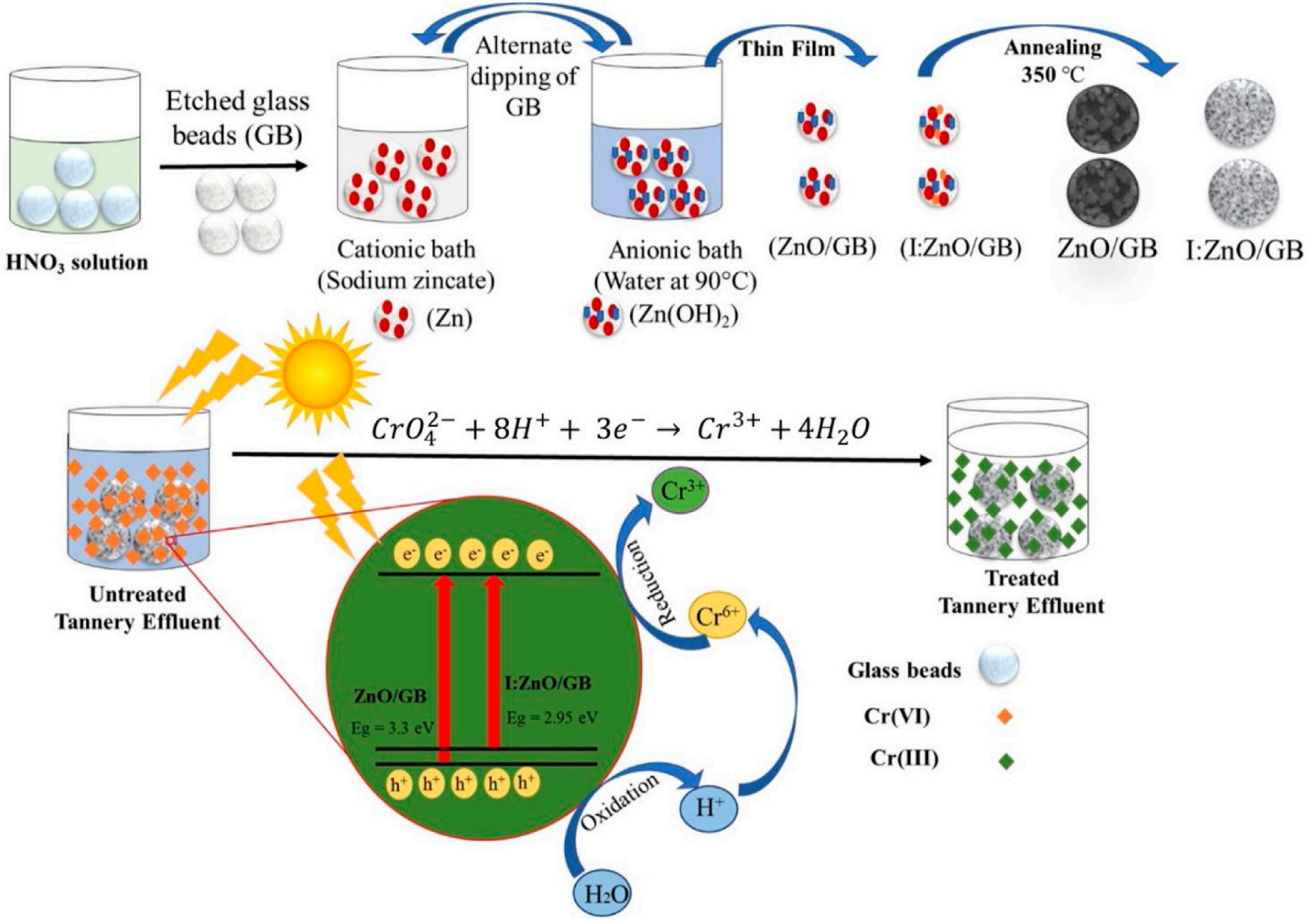


## Introduction

Water pollution is increasing day by day due to industrial, municipal, and agricultural effluents that are discharged without any treatment into the water bodies (Sudarsan and Krishnamoorthy, 2018; Dong et al., 2019). These effluents contain pollutants such as detergents, heavy metals, fertilizers, dyes, and residues of domestic, agricultural, and industrial waste material which are contaminating clean water (Fernández et al., 2010; Miao et al., 2018; Khezrianjoo et al., 2019; Rafiq et al., 2019; Ashar et al., 2020; Truong et al., 2020; Mohsin et al., 2021b). In effluent, heavy metals are the major environmental pollutants that are adversely affecting the ecological food chain (Ghezali et al., 2018; Ibisi and Asoluka, 2018; Mansouri et al., 2018; Alkherraz et al., 2020). The most common heavy metals present in effluent are chromium (Cruz-Zavala et al., 2016), lead (Pb), copper (Borges et al.), cadmium (Cd), zinc (Khan M. I. et al., 2020), nickel (Ni), mercury (Oskoei et al., 2016), and silver (Ag). Among them, Cr with oxidation state (VI), i.e., Cr(VI) is highly toxic because it can easily penetrate through the cell membrane without any hindrance, which ultimately cause oxidation of biological molecules leading to death of living cells (Majolagbe et al., 2016; Djehaf et al., 2017; Abbas et al., 2018; Iwuoha and Akinseye, 2019). When Cr (VI) is present above the permissible limit, it causes serious environmental problems such as reduction in vegetative growth of plant and its yield, eventually causing plant death. Since Cr (VI) is strongly phytotoxic, it eventually has led to food insecurity and scarcity (Iqbal, 2016; Abbas et al., 2018). Humans upon consuming plants containing Cr(VI) will suffer from diseases such as lung tumors, diarrhea, headache, and nausea. Therefore, effluent containing Cr(VI) needs to be treated before discharge for the survival of living beings (Sezgin et al., 2016). Until now, many physical, chemical, and biological processes have been tried for effluent treatment. However, socioeconomic and environmental issues associated with these technologies are a huge hurdle in application of these methodologies for Cr(VI)-containing effluent treatment (Ukpaka, 2016; Legrouri et al., 2017; Palutoglu et al., 2018; Sasmaz et al., 2018; Khan N. A. et al., 2020).

Advanced oxidation processes (AOPs) are one of the appropriate technologies for removal of pollutants from effluent, and photocatalytic removal of heavy metals is very fast as compared to other AOPs (Iqbal and Bhatti, 2015; Jamal et al., 2015; Wu et al., 2020; Ishaq et al., 2021). So far, setup for the reduction of heavy metals and decomposition of organic pollutants using optimized combinations of oxidants, catalyst loading time, and irradiation time have been developed. Mechanistically, photocatalysis is a photo-induced redox reaction that is accelerated in the presence of a photocatalyst due to generation of electrons and hole pairs and ultimate production of highly reactive free radicals (Dong et al., 2021a; Dong et al., 2021b). Photocatalytic activity (PCA) of the photocatalysts can be enhanced by doping with metal and nonmetal ions resulting in the variation in optical properties (Ata et al., 2018). The enhancement in PCA of ZnO by red shifting the optical response toward the visible region of the solar spectrum is a favorable effect (Mohsin et al., 2021a). Extensive research is being focused on decreasing bandgap energy for excitation of semiconductor photocatalysts, in order to avail a larger portion of the visible region of the solar spectrum. Among several methods being explored to improve visible light harvesting through the shifting of absorption edge of semiconductors to longer wavelengths, the successful one is metal or nonmetal ion doping (Umar et al., 2015; Ashar et al., 2020; Dong et al., 2020; Mohsin et al., 2020).

Various semiconductor metal oxides, such as TiO_2_, ZnO, ZnS, and WO_3_, have been used as a photocatalyst on exposure to suitable light source (Ashar et al., 2016; Vaiano and Iervolino, 2019; Arshad et al., 2020; Bokhari et al., 2020; Inderyas et al., 2020; Parveen et al., 2020; Vallejo et al., 2020; Bhutta et al., 2021). However, ZnO with a tunable gap of ∼3.3 eV and high surface to volume ratio with low toxicity has suitable characteristics to be used as a photocatalyst. Its bandgap can be reduced by doping with different metals and nonmetals by replacing Zn and O atoms in the ZnO lattice (Ata et al., 2018; Borovinskaya et al., 2019; Inderyas et al., 2020). Until now, anionic doping of ZnO has not been as widely studied as that of cationic doping. Specifically, halogens as an effective n-type dopant element has not been reported much for photocatalytic investigations. Doping of ZnO with halogens in VII column (F, Cl, I, and Br) involves replacement of the oxygen in the ZnO lattice (Rousset et al., 2009). It has been examined that 2D structures of photocatalysts have a positive impact on PCA of ZnO because of a high content of surface defects as compared to other morphologies such as nanorods (Liu et al., 2009). The previous reports based on photocatalysis of organic pollutants include the high PCA of iodine-doped TiO_2_ on visible light irradiation (Liu et al., 2009). Moreover, immobilization of the nanophotocatalyst renders its recovery easy, from the reaction mixture to overcome the predicament of the slurry reactor. The ZnO photocatalyst can be adhered to miscellaneous substrates such as glass beads/glass slabs, ceramics, sand, activated carbons (ACs), stainless steel, and inorganic materials that allow the continuous reuse (Borges et al., 2016; Ahmad et al., 2017).

Among different materials, glass substrate is a quite promising material to be used as a support because of its distinctive properties, i.e., higher surface area, superior adsorption, reduction in charge recombination, and transparency of glass are retained intact even after coating of the photocatalyst that will not disturb light penetration (photon flux) and rate of photocatalysis (Amir et al., 2016; González-Rodríguez et al., 2020). Moreover, the shape and texture of the substrate also affect the available surface area for interaction with the pollutant (Dong et al., 2021a; Dong et al., 2021b). The cost of locally available glass beads is only 0.075 US dollar/12 piece (market price); therefore, utilizing glass beads as a substrate will make this technique both economically and potentially feasible to be applied at large scale. Photocatalysts can be deposited over the heterogenous support by chemical and physical methods. Among thin film deposition methods, the double dip technique also named as successive ionic layer adsorption and reaction (SILAR) from aqueous solutions is the simplest and the most economical one (Mohsin et al., 2020; Ashar et al., 2021).

Keeping these facts in consideration, the glass beads were used as a suitable support for immobilization of ZnO and iodine-doped ZnO to fabricate ZnO/GB and I:ZnO/GB hybrid that is used for the photoreduction of Cr(VI) heavy metal from industrial effluent. I:ZnO nano-bricks were grafted onto the glass beads through the SILAR method. Operational parameters, i.e., initial Cr(VI) concentration, pH, and sunlight exposure time to study photoreduction of Cr(VI) were optimized by response surface methodology. Employment of our designed I:ZnO/GB hybrid as a photocatalyst has introduced a novel cost-effective solar photocatalytic reactor for simultaneous adsorption and photoreduction of heavy metals present in effluent efficiently. The treated effluent containing permissible amount of Cr (0.1 ppm) is reusable for irrigation of crops and industrial operations.

## Materials and Methods

### Chemicals

Zinc sulfate heptahydrate (ZnSO_4._7H_2_O), potassium iodide (KI), sodium hydroxide (NaOH), sulfuric acid (H_2_SO_4_), hydrochloric acid (HCl), and 1, 5-diphenylcarbazide were purchased from Sigma-Aldrich and used without any further purification during research.

### Grafting of Intrinsic and Iodine-Doped ZnO

**FIGURE 1:**
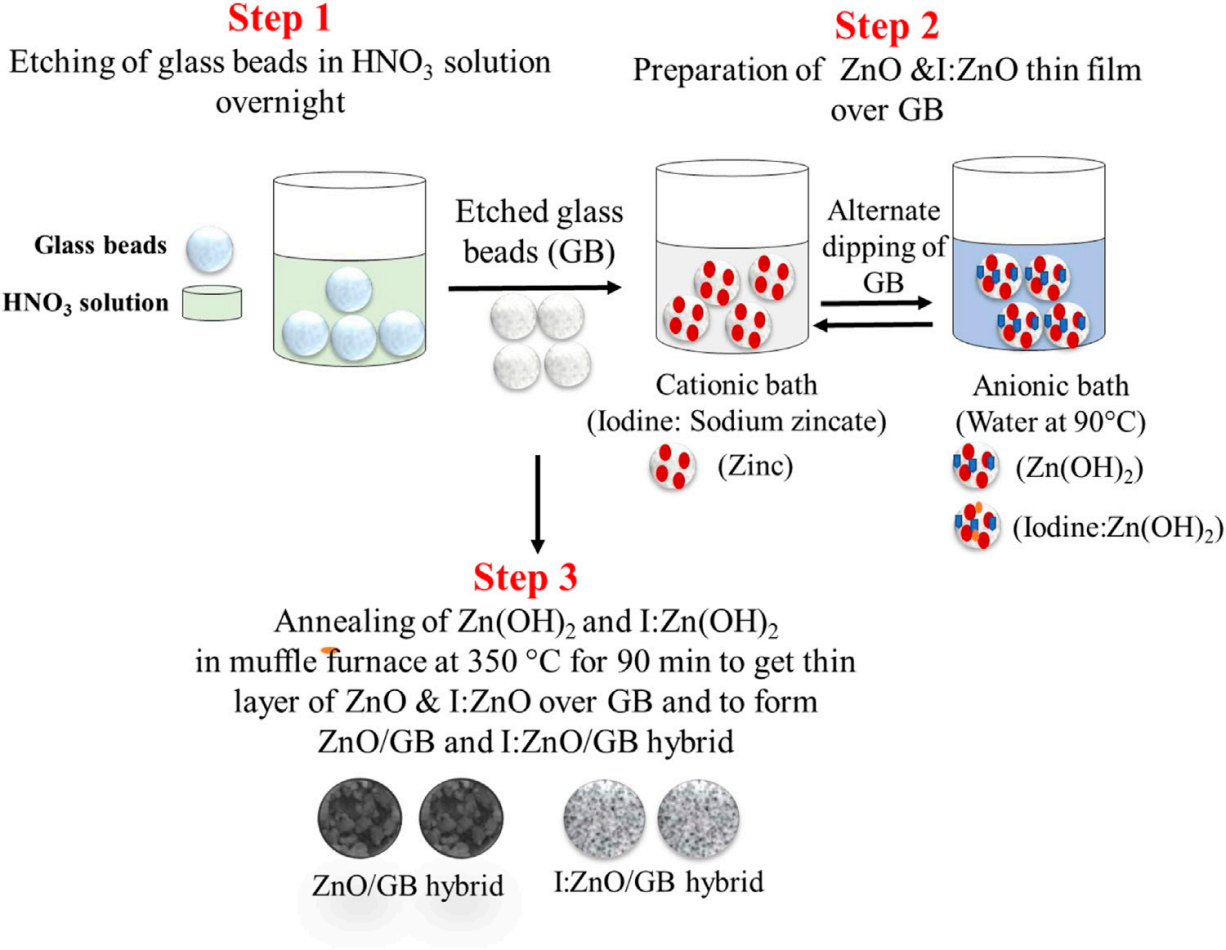
Schematic diagram of fabrication of ZnO/GB and I:ZnO/GB hybrid by the SILAR method.

In continuation of previous studies (Zhao et al., 2013; Zheng et al., 2017), intrinsic ZnO and iodine-doped ZnO were grafted onto the glass beads through the SILAR method. Initially, glass beads were etched by dipping in 30% HNO_3_ solution overnight at 30°C, followed by washing with ethanol and deionized water. Later on, cationic solution was prepared by mixing ZnSO_4_.7H_2_O (0.08 M) and NaOH (0.2 M) in a 1:10 V/V ratio, i.e., 10 ml of ZnSO_4_.7H_2_O solution into which 100 ml NaOH was added for ZnO/GB. Additionally, 4% of KI (3 mM) was added to the cationic solution as a source of iodine dopant for I:ZnO/GB (Thomas et al., 2016). Water was used as an anionic solution, and its temperature was set near its boiling point (90°C) using a water bath. Finally, the glass beads were immersed into cationic bath and left for 30 s at room temperature. Then, beads were removed from cationic bath and immersed into 100 ml anionic bath for 30 s at 90°C. After completion of one cycle of alternative dipping in cationic and anionic bath, glass beads were air-dried for 10 s. This process was repeated for 20 cycles for both (ZnO and I:ZnO), followed by drying and annealing at 350°C for 90 min in a muffle furnace (Ashar et al., 2021). This process ultimately grafted the thin film of ZnO and I:ZnO onto the glass beads as a substrate, resulting in the development of solar photocatalysts. Schematic representation for fabrication of ZnO/GB and I:ZnO/GB hybrid is given in Figure 1.

### Characterization of ZnO/GB and I:ZnO/GB Hybrid

For characterization of the fabricated material, ZnO/GB and I:ZnO/GB hybrid were scrapped finely from the surface of functionalized glass beads and used for characterization. The crystallographic structure of intrinsic ZnO and I:ZnO was examined by using an X-ray diffractometer (JEOL JDX-3532 diffractometer) utilizing CuKα irradiation (*λ* = 1.54 Å). Surface morphology was determined using a scanning electron microscope (Quanta 2,500 FEG) and transmission electron microscope (TEM) (JEOL 2M 2100). Elemental analysis was performed by energy dispersive X-ray (EDX) (INCA Oxford Instruments UK). Diffused reflectance spectroscopy (DRS) was measured and used to determine bandgap of ZnO and I:ZnO (Perkin Elmer Lambda 1,050, United States). For thermal stability assessment, TGA of intrinsic ZnO and I:ZnO was carried out (PerkinElmer thermal analyzer).

### Photocatalytic Reduction of Cr(VI) by ZnO/GB and I:ZnO/GB Hybrid

The photocatalytic activity of fabricated materials ZnO/GB and I:ZnO/GB was evaluated by photocatalytic reduction of Cr(VI). The batch experiments were performed at conditions optimized by response surface methodology using I:ZnO/GB hybrid. All experiments were performed in borosilicate cubical containers of 10 × 10 × 4 cm dimensions with a working volume of 100 ml of Cr(VI) that was brought in contact with ZnO/GB hybrid (44 mm size, 10% filling ratio) under direct natural sunlight (solar flux ranged from 600 to 650 KWh/m^2^). Reduction of Cr(VI) to Cr(III) was determined by change in intensity of color using a UV/visible spectrophotometer (CECIL 7,200, Germany). The wavelength of maximum absorbance of Cr(VI) is λ_max_ = 474 nm, whereas λ_max_ for Cr(III) is 353 nm. The calibration curve was drawn to find out the remaining concentration (unreduced) of Cr(VI), followed by estimation of percentage reduction of Cr(VI) using Eq. 1.

$$\mathrm{Reduction}(\%) =\;\;\frac{{\text{C}}_{0-}{\text{C}}_{\text{f}}}{{\text{C}}_{0}}\times 100,$$ (1)

where, C_0_: initial concentration of Cr(VI) (ppm); C_f_: final concentration of Cr(VI) (ppm). The similar method has been frequently used for photocatalytic degradation of dyes (Saravanan et al., 2016).

### Optimization of Parameters by Response Surface Methodology

Response surface methodology (RSM) was applied with the help of Design Expert 7.0.0 software to optimize three reaction parameters such as pH (5–9), initial concentration of standard Cr(VI) (10–50 ppm), and irradiation time (1–5 h) to get maximum % reduction of Cr(VI). The central composite design (CCD) was applied to investigate the combined effect of the four independent variables by 20 runs of experiments, and % reduction of Cr(VI) was taken as response. Later on, the samples of real tannery effluent were treated with I:ZnO/GB hybrid under optimized conditions to study the reduction of Cr(VI) to Cr(III).

### Evaluation of Extent of Reduction of Cr(VI)

To determine the extent of reduction of Cr(VI) to Cr(III), UV/visible spectroscopy of untreated and treated tannery waste and complexes of Cr in both oxidation states was carried out. The kinetic study of the photocatalytic reduction reaction was also investigated for ZnO/GB and I:ZnO/GB hybrid. Total % removal of Cr was determined by atomic absorption spectroscopy (AAS) (Thermo Electron Co., Solar M Series).

### Complexation of Cr(VI) With 1, 5-Diphenylcarbazide

To detect presence of chromium in two different oxidation states in real tannery effluent, the complexation was performed using a complexing reagent. Tannery effluent contained hexavalent chromium which formed a complex with 1, 5-diphenylcarbazide (1, 5 –DPC). The complexing reagent was prepared by dissolving 0.5 g of 1, 5-DPC in 100 ml of acetone, with further dilution with distilled water. A sample of tannery effluent prepared in different concentrations was used for solar photocatalytic reduction of Cr(VI). To prepare extraction solution, 0.1% potassium permanganate was dissolved in water until pink color was obtained. After mixing these reagents in tannery effluent, 4 ml of sulfuric acid was added till the pH obtained was 2. Then, 1, 5-DPC solution (2 ml) was added into the tannery effluent before and after solar photocatalytic treatment (Lace et al., 2019).

## Result and Discussion

### Structural Characterization of ZnO and I:ZnO

**FIGURE 2:**
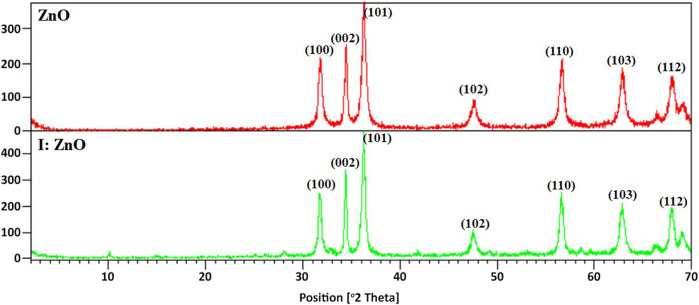
Diffractograms of ZnO and I:ZnO photocatalysts obtained from ZnO/GB and I:ZnO/GB hybrid.

Crystallinity of prepared ZnO and I:ZnO hybrid was confirmed by XRD diffractogram obtained for ZnO and I:ZnO (Figure 2). Highly crystalline intrinsic ZnO and I-doped ZnO have been synthesized, as obvious from the XRD pattern. ZnO and I:ZnO hybrid were highly pure since no peaks of impurities were observed in the XRD pattern (Figure 2). The 2Ɵ values 31. 32°, 34.35°, and 36.10° classified the Miller indices (100), (002), and (101), respectively, that confirmed the prepared product to be ZnO and I:ZnO (JCPDS No. 36–1,451). It is clear from Figure 2 (101) that the peak with maximum intensity appeared at 2Ɵ = 36.10°; its intensity is enhanced by doping with iodine which demonstrated that the synthesized iodine-doped ZnO sample also possessed the wurtzite hexagonal structure but with higher crystallinity (Zheng et al., 2017). The strong diffractive peaks indexed at (100), (002), and (101) have shown a slight shift toward lower (2θ) values than those of intrinsic ZnO, signifying the optimized doping of I in the ZnO crystal structure (Mahmood et al., 201). Since dopants should not contribute significantly to peak shifts, a slight difference in lattice parameters had occurred. This issue is presumably related to the difference in ionic sizes of I (0.206 nm) and oxygen (0.132 nm) ions. The diffractograms of I-doped ZnO have shown a very slight decrease in “a” and “c” lattice parameters which is in accordance with previous research and can be explained as a result of the partial substitution of oxygen ions in the system, respectively (Thomas et al., 2016).

The greater peak height at (002) than (100) observed in the case of I:ZnO indicated that thicker 2D structures were expected to be formed on iodine doping. The average particle size of ZnO and I:ZnO was calculated by Debye–Scherrer’s formula (D = kλ/βCosθ). The results obtained from Debye–Scherrer’s formula showed that the average crystallite size of ZnO and I:ZnO was 20.34 and 26.23 nm, respectively. Since the crystallite size of intrinsic ZnO was smaller, therefore, the general trend of an increase in the crystallite size can be attributed to I doping. The similar trend has been noticed by another group who has reported the increase in crystallite size from 18 to 35 nm on I doping. The reason behind this fact is extension in the grain boundary for larger grain sizes and substitution of iodine ions at the site of oxygen ions (Bindu and Thomas, 2014).

**FIGURE 3:**
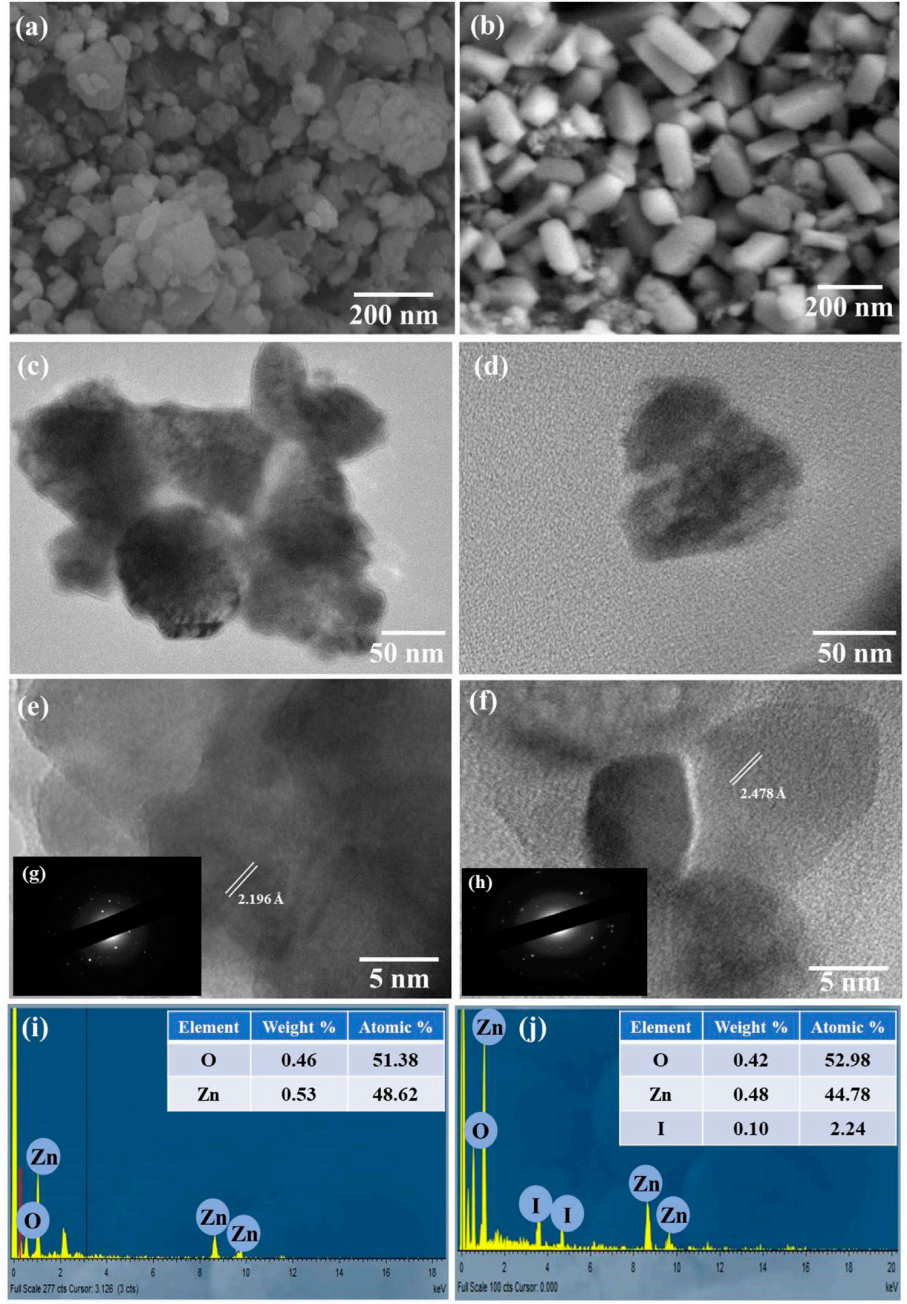
SEM images of **(A)** ZnO nanodiscs, **(B)** I:ZnO nano-bricks, **(C)** TEM image of ZnO nanodiscs, **(D)** TEM image of I:ZnO nano-bricks, **(E)** HRTEM of ZnO nanodiscs, **(F)** HRTEM of I:ZnO nano-bricks, **(G)** SAED of ZnO nanodiscs, **(H)** SAED of I:ZnO nano-bricks, **(I)** EDX analysis of ZnO nanodiscs, and **(J)** EDX analysis of I:ZnO nano-bricks.

Surface morphology of pure ZnO and I:ZnO was determined by SEM and TEM (Figure 3); SEM micrographs of intrinsic ZnO expressed the mono-modal discs having oval shape which appeared to be agglomerated. However, the micrograph of I:ZnO exhibited that particles adopted the shape of bricks after doping ZnO with iodine, so they are named as nano-bricks. These nanostructures of hexagonal shape have edges and kinks, developed at the surface which can be observed clearly. An increased number of active sites for surface solar photocatalysis have been created in the lattice of I:ZnO containing excess of exposed oxygen vacancies, crystal defects, and high content of surface hydroxylation. The higher expected PCA of I:ZnO attributes to the flattened and kinked exposed surfaces of nano-bricks adhered to glass beads (Del Gobbo et al., 2019).

TEM of intrinsic ZnO discs and I:ZnO nano-bricks is shown in the Figures 3C,D. The average width of the oval disc was measured by ImageJ software as 48 nm, length 123 nm, and thickness 21 nm. A few bricks were captured through TEM; the micrograph supported the results of SEM. The thickness of bricks was measured as 27 nm, width 50 nm, and length 95 nm. The tapering ends, edges, and kinks appearing in bricks are considered active sites for photocatalysis (Ashar et al., 2020); so, it was expected from 2D-brick-like structures to exhibit high rate of reduction of Cr(VI).

HRTEM of both samples has exhibited that monocrystalline structures were synthesized (Figures 3E,F). The high resolution TEM images at 400 KX exhibited the continuous crystalline planes indicating the monocrystalline nature of I:ZnO nano-bricks. The results obtained by SAED also supported this fact, and d-spacing was found to be 2.478 Å in the crystal of I:ZnO, greater than that of intrinsic ZnO (2.196 Å) (Figures 3G,H). The variation in morphology and d-spacing further confirmed doping of ZnO with iodine. The results were found concurrent to previous investigations (Wang et al., 2016).

Furthermore, the elemental composition and purity of the synthesized sample was confirmed by EDX analysis (Figure 3). It is quite obvious from the EDX spectrum (Figures 3I,J) that ZnO nanodiscs and I:ZnO nano-bricks contain Zn, O, and I and absence of any other element is the proof of high purity of the fabricated ZnO and I:ZnO. For intrinsic ZnO, Zn in higher weight percent (0.46%) and atomic percent (26.69%) is present than those of O (32.73% atomic and 0.53% weight). On the other hand, I:ZnO contained 67.27% atomic and 1.14% weight of Zn. The concentration of Zn is comparatively higher than that of O in the lattice of I:ZnO, indicating the presence of high content of oxygen vacancies. However, the higher atomic percent (46.82%) of oxygen in I:ZnO than that of intrinsic ZnO revealed the high surface content of atomic oxygen, including bonded and free atomic oxygen. The small amount of iodine, i.e., 0.10 weight% and 2.24 atomic% have confirmed the negligible concentration of iodine in I:ZnO as a dopant. The results obtained were in agreement with XRD as no peak for iodine appeared in the diffractogram.

**FIGURE 4:**
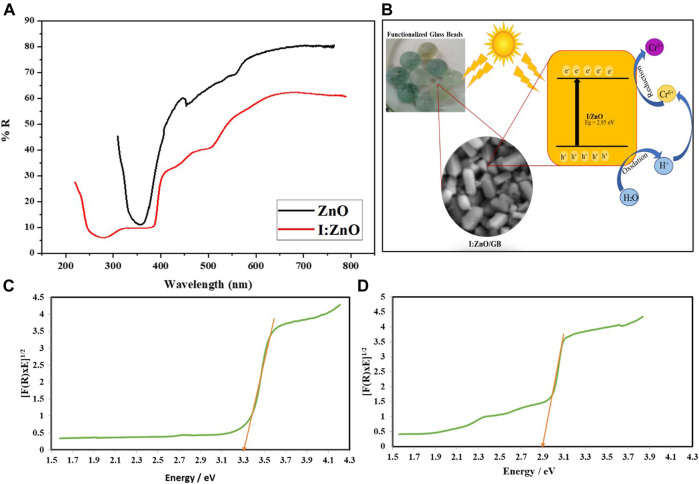
**(A)** Diffused reflectance spectrum indicating the higher absorption of sunlight by I:ZnO/GB than ZnO/GB; **(B)** schematic diagram of the solar photocatalytic reduction reaction carried out by I:ZnO grafted on glass beads; **(C)** bandgap of ZnO/GB; and **(D)** bandgap of I:ZnO/GB.

Furthermore, the optical properties such as diffused reflectance spectra and bandgap of ZnO and I:ZnO were also determined as shown in Figure 4. The diffused reflectance spectrum delineated that more than 70% of sunlight, in case of ZnO, and less than 60%, in case of I:ZnO, was reflected indicating significantly higher sunlight harvesting tendency in case of I:ZnO (Figure 4A). The bandgap energy depends upon the crystal structure and electronic configuration of the band, while the bandgap energy affects optical properties of the photocatalyst by directly influencing the photocatalytic activity (Dong et al., 2020; Jilani et al., 2020). The schematic diagram has been drawn to show the greater tendency of absorption of sunlight in the visible region by I:ZnO due to lesser bandgap energy (Figure 4B). The bandgap was determined by the Kubelka–Munk plot with the data obtained from diffused reflectance spectroscopy (Ashar et al., 2020). The bandgap for ZnO (Figure 4C) and I:ZnO (Figure 4D) was 3.30 and 2.95 eV, respectively. It can be concluded that the decrease in bandgap on doping of ZnO with iodine caused the increase in sunlight harvesting capability of the photocatalyst, in a visible region. The photocatalytic reduction of Cr in the presence of I:ZnO can be attributed to a higher rate of generation of e^−^h^+^ pair on exposure to sunlight.

Considering the results of TGA for both synthesized samples, the initial weight loss for both intrinsic and iodine-doped ZnO, observed between 100 and 500°C, was due to the physically or/and chemically adsorbed hydroxide and water molecules by evaporation. In case of intrinsic ZnO discs, the first gradual weight loss of 2.34% took place from 180–220°C, then a greater rate of weight loss of 9.73% was noticed in the temperature range of 230–280°C due to the evaporation of surface-absorbed water indicating the decomposition of Zn(OH)_2_. The third weight loss of 11.15% occurred in the temperature range of 300–400°C due to complete loss of water from the sample. The stability in weight has been exhibited from 350 to 600°C, while the slow weight loss continued up to 800°C.

On the other hand, I:ZnO nanobricks were found to be thermally more stable; I:ZnO started losing weight (1.18%) from 150 to 200°C and then a drop in weight of 7.34% took place between 260–300°C. After that, the doped sample attained the stability up to 700°C. Doping of ZnO with cations and anions enhances the thermal stability of the photocatalyst since this fact has also been explained by other researchers (Arshad et al., 2018). The plateau observed for the iodine-doped ZnO sample exhibited lower total weight loss confirming the greater thermal stability of the doped sample than the intrinsic ZnO sample. It can further be attributed to the incorporation of iodide ions into some of the oxygen lattice sites of ZnO (Sajjad et al., 2018). Morphology of the nanostructures also plays a role in thermal stability. Comparatively, the larger grain boundary and greater thickness of the particles enhance the thermal stability. It can be conferred that thicker I:ZnO nano-bricks with greater crystallite size have shown higher thermal stability than intrinsic ZnO nanodiscs (Moharram et al., 2014; Raza et al., 2016; Mohan et al., 2020).

### Determination of Photocatalytic Activity

The fabricated photocatalysts ZnO/GB and I:ZnO/GB were used for reduction of Cr(VI) to Cr(III) under natural sunlight in the batch reactions. The standard solutions of Cr(VI) were used as a probe for solar photocatalytic reduction in the range of 10–50 ppm. The solutions were exposed to natural sunlight in an open air reactor on adjusting pH with a working volume of 100 ml.

#### Optimization of Reaction Parameters for Reduction of Cr(VI) by Response Surface Methodology

RSM was applied to optimize three different parameters which were selected as reaction variables, while the reduction of Cr(VI) was the output response. The percentage (%) reduction was taken as response, while the values of variables X_1_, X_2_, and X_3_ were used to indicate pH, irradiation time of sunlight, and initial concentration of Cr(VI), respectively.

#### Interactive Effects of Factors Affecting Photocatalytic Degradation

**FIGURE 5:**
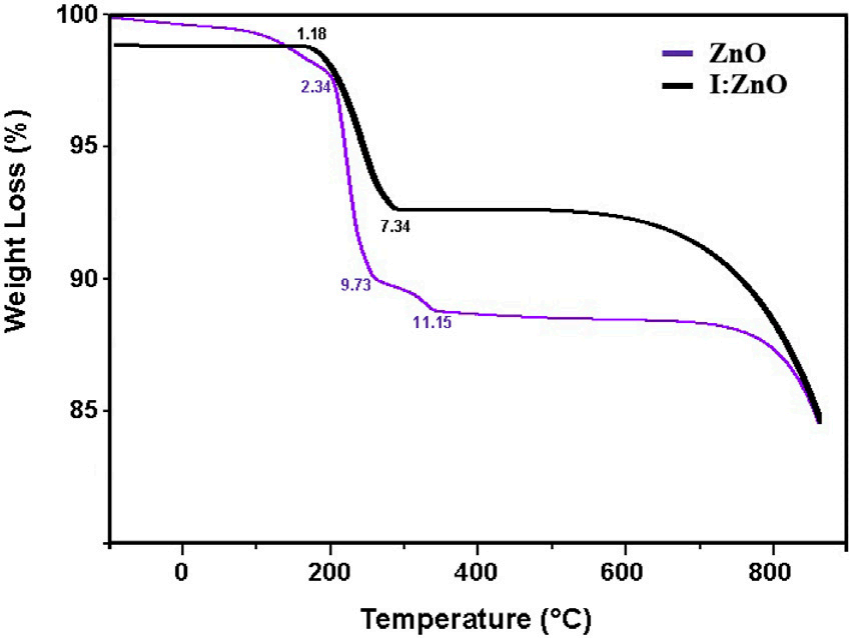
TGA analysis of fabricated ZnO/GB and I:ZnO/GB.

**FIGURE 6:**
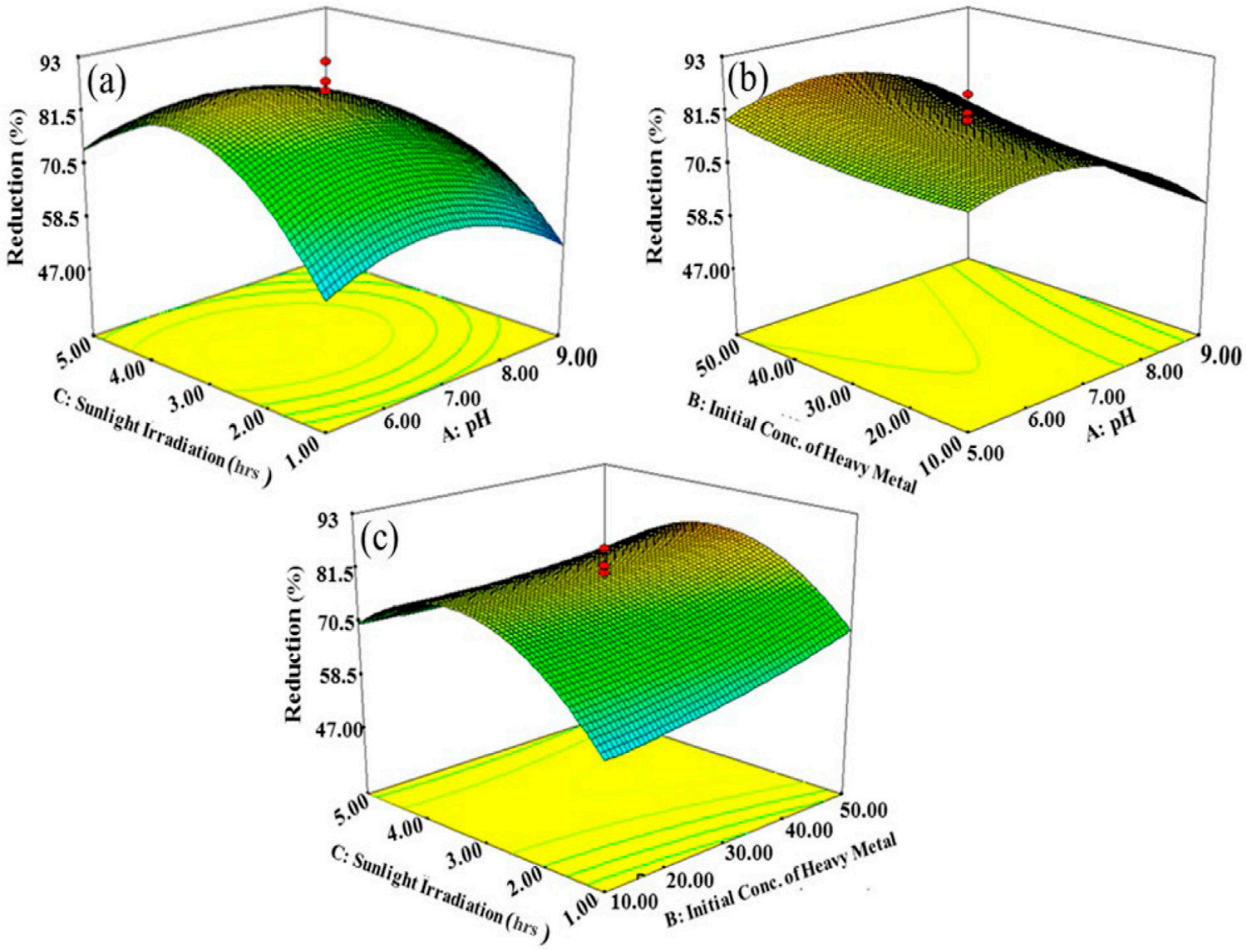
Interactive effects. **(A)** Sunlight irradiation time and pH, **(B)** initial concentration of Cr(VI) and pH, and **(C)** initial concentration of Cr (VI) and sunlight irradiation time.

The interactive effects of variables affecting photocatalytic reduction of Cr(VI) in terms of 3D surface plots of RSM modeling are shown in Figure 6. There are number of factors that govern the photocatalytic reduction of Cr(VI); among these factors, the interactive effect of pH and irradiation time is represented in Figure 5A which gives the direct relation between pH and irradiation time of sunlight. Photocatalytic reduction of Cr(VI) was enhanced by increasing irradiation time from 2 to 4 h. Another important factor is pH, which affects the reduction rate of Cr(VI) by controlling surface charge of the photocatalyst during the photocatalytic reaction. The pH of the reaction mixture not only affects the chemistry of metal ion but also the surface charge of the photocatalyst. The rate of photocatalysis first increased with an increase in pH, but at basic pH it has been observed to decrease due to the occupancy of active sites by hydroxylated complexes of the metal ions (Yuvaraja et al., 2018). At low pH values, H^+^ covered the surface of the photocatalyst resulting in less concentration of Cr(VI) approaching the active sites because of electrostatic repulsion. On increasing pH values, the surface became negatively charged which accelerated the adsorption of metal ions, reaching the maximum at pH 7.0. Thus, the optimum pH was selected to be 7.0 for promoting the rate of photocatalytic reduction of Cr(VI) to Cr(III), also confirmed from the 3D RSM plot (Figure 6B).

The solution pH and irradiation time of sunlight have also shown interactive effect; varying one variable also affects the other variable that ultimately has a great impact on the reduction of Cr(VI) (Figure 6C). When the pH and irradiation time were increased, then the removal of Cr(VI) from tannery effluent was also increased indicating the direct relation. But, the rate of degradation increased up to a certain limit, and then it became constant as it started decreasing upon further increasing pH and irradiation time. Moreover, upon long time exposure to solar radiations, byproducts get accumulated on the active sites of the catalyst, resulting in the deactivation of photocatalyst causing the decrease in the rate of photoreduction of Cr(VI) (Qureshi et al., 2019).

Initial concentration of Cr(VI) is another factor which greatly affects the photocatalytic extent of reduction (Figures 6B,C). An increase in the initial concentration of Cr(VI) decreases the removal efficiency which is due to shielding of sunlight by Cr(VI) to reach the photocatalyst that decreases the Cr(VI) reduction rate. The experimental results represented by RSM justify the inverse relation between Cr(VI) concentration and reduction efficiency. The photocatalytic reduction of Cr(VI) was maximum at 30 ppm, and it decreased on increasing initial concentration of Cr(VI). The interactive effect of initial concentration of Cr(VI) and irradiation time is given in Figure 6C, according to the results obtained by increasing irradiation time of sunlight reduction efficiency. However, after optimum irradiation time of 3 h and increased initial concentration of Cr(VI), photocatalytic reduction decreased. Although, on increasing the initial concentration of Cr(VI) from 10 to 30 ppm, the reduction efficiency increased, but after that it decreased on further increasing the concentration of heavy metal. The complete coverage of active sites with Cr(VI) caused the decrease in the rate of reduction on further increasing the initial concentration of Cr(VI). The 3D response surface plots of RSM revealed that maximum photoreduction of Cr(VI) to Cr(III) can be achieved at optimum reaction conditions such as neutral pH (7), 3 h sunlight exposure time, and 30 ppm of Cr(VI) initial concentration.

The reaction variables have an significant interactive effect on the response surface as given in the ANOVA table. The lower value of prob. F ˃ 0.0047 indicated the fitting of the rotatable central composite design (RCCD) applied. In addition, the Pred. R^2^ = 0.8680 is in agreement with Adj. R^2^ = 0.9395 which shows that the model is highly significant, and insignificant lack of the fit model shows high predictability (Table 1).

**TABLE 1:** ANOVA for the response surface quadratic model.

Source	Sum of squares	Df	Mean square	F value	*p*-value probe > F	
Model	4,299.68	9	477.74	3.30	0.0383	Significant
A-pH	295.67	1	295.67	2.04	0.1834	
B-initial concentration of heavy metal	516.65	1	516.65	3.57	0.0882	
C-sunlight irradiation time	715.01	1	715.01	4.94	0.0505	
AB	8.00	1	8.00	0.055	0.8189	
AC	0.50	1	0.50	3.455E-003	0.9543	
BC	128.00	1	128.00	0.88	0.3692	
A^2^	1,615.73	1	1,615.73	11.16	0.0075	
B^2^	716.88	1	716.88	4.95	0.0502	
C^2^	790.55	1	790.55	5.46	0.0415	
Residual	1,447.37	10	144.74			
Lack of fit	1,443.37	5	288.67	360.84	˂0.0001	Nonsignificant
Pure error	4.00	5	0.80			
Core total	5,747.05	19				

### Complexation of Cr(VI) and Cr(III) With 1, 5-Diphenylcarbazide

**FIGURE 7:**
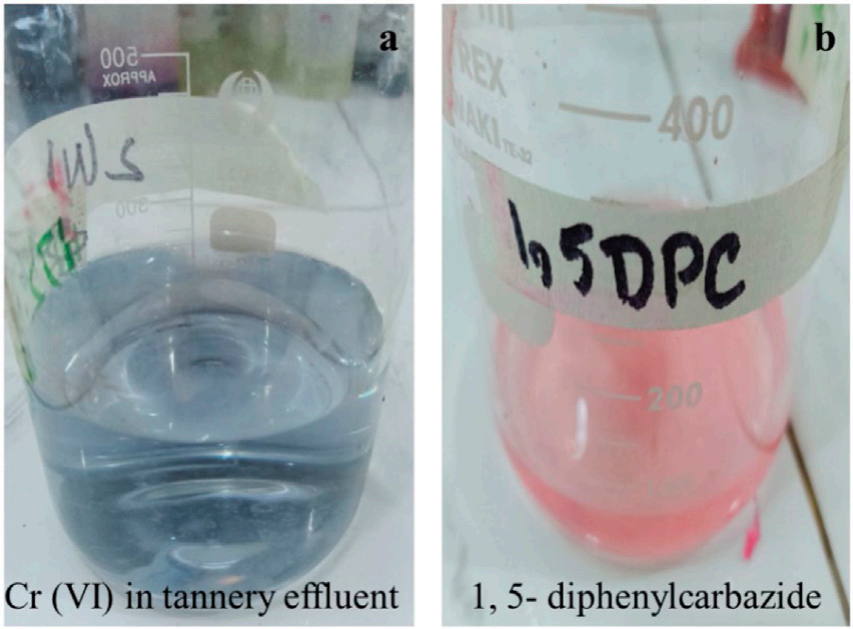
Formation of complex of Cr(VI) with 1, 5-DPC. **(A)** Tannery effluent before solar photocatalytic reduction of Cr(VI); **(B)** complex of Cr(VI) with 1, 5-DPC giving pink color.

Photocatalytic reduction of Cr(VI) to Cr(III) was confirmed by the formation of complexes of Cr with 1, 5-DPC. The pinkish-violet-colored complex of Cr(VI) was formed rapidly on reaction with the complexing reagent (Figure 7A,B). A UV/visible spectrophotometer was used for determination of the hexavalent chromium complex with 1, 5-DPC, and a peak appeared at 547 nm (Onchoke and Sasu, 2016).

**FIGURE 8:**
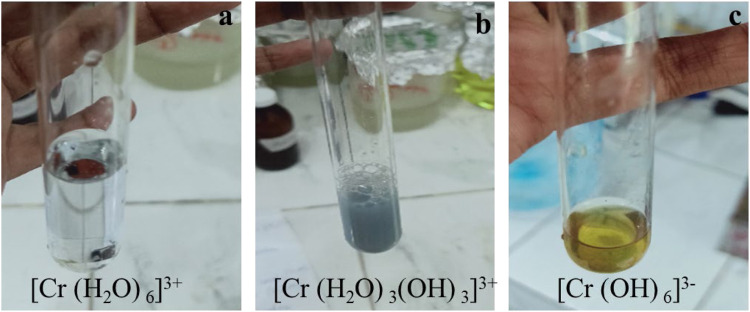
Complexes of Cr (III) after solar photocatalytic reduction. **(A)** [Cr(H_2_O)_6_]^3+^ **(B)** [Cr(H_2_O)_3_(OH)_3_]^3+^ **(C)** [Cr(OH)_6_]^3-^.

In tannery effluent, Cr(VI) was reduced to Cr(III) by solar photocatalysis using I:ZnO/GB. Then, after addition of alcohol and acid, green color of hexa-aqua-chromium (III) ion Cr(H_2_O)^3+^_6_ appeared due to a complex formed by Cr(III) in aqueous solution (Lennartson, 2014) (Figure 8). Thus, it is clear from results that Cr(III) forms octahedral Cr(H_2_O)^3+^_6_, {Cr(H_2_O)_3_(OH)_3_}, and {Cr(OH)_6_}^3-^ complex ions in the aqueous solution (Theopold, 2006). The reduction of Cr(VI) to Cr(III) in standard solutions and tannery effluent was qualitatively determined before and after the solar photocatalytic reaction was carried out in an open air reactor under natural sunlight.

### Evaluation of Photocatalytic Reduction of Cr(VI) to Cr(III) Using I:ZnO/GB Hybrid

**FIGURE 9:**
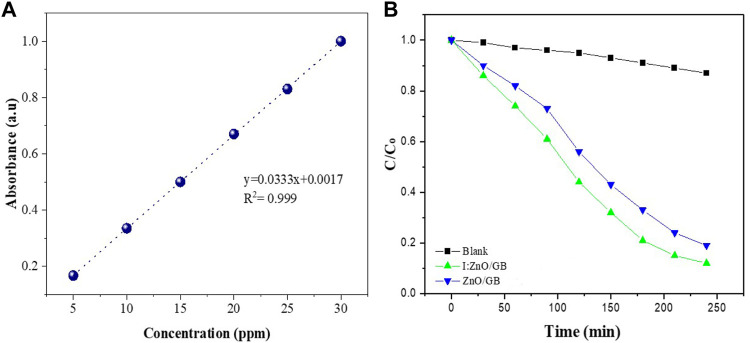
**(A)** Calibration curve drawn using a UV–vis spectrophotometer for standards of Cr (VI) and **(B)** the comparative analysis of photocatalytic activity of blank, ZnO/GB hybrid, and I:ZnO/GB hybrid for reduction of Cr (VI) as a function of time.

The photocatalytic activity (PCA) of I:ZnO/GB hybrid was compared with the blank sample as a function of time using standards of Cr(VI) (Figure 9A). As indicated in the graph (Figure 9B), I:ZnO/GB hybrid showed better efficiency for solar photocatalytic reduction of Cr(VI) than ZnO/GB. The change in concentration has been reported in terms of C/C_o_ (final concentration of Cr(VI)/initial concentration of Cr(VI)) with the passage of time. It can be clearly seen that in case of blank variation in C/C_o_ with respect to time is almost negligible. While in case of I:ZnO/GB hybrid, photoreduction of Cr(VI) to Cr(III) was much more efficient than ZnO/GB.

### Photocatalytic Reduction of Cr(VI) toCr(III) in Real Tannery Effluent

**FIGURE10:**
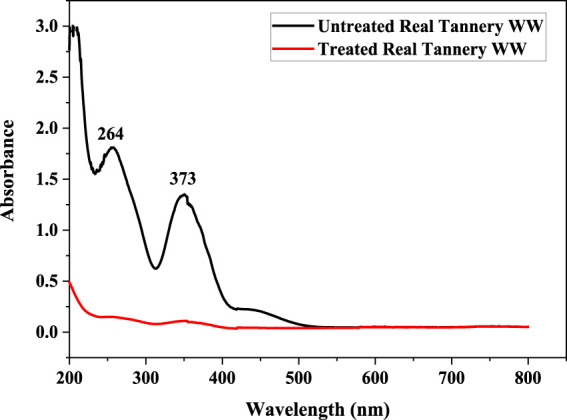
UV/vis spectra of tannery effluent before and after solar photocatalytic treatment using I:ZnO/GB hybrid.

Photocatalytic reduction of Cr(VI) in the tannery effluent sample was carried out under conditions optimized by RSM (pH: 7, Irradiation time: 4 h). The UV/Vis spectrophotometry was used for the confirmation of photocatalytic reduction of Cr(VI) in the sample. The spectrophotometric analysis of Cr(VI) in tannery effluent showed that the sample contained Cr(VI) before solar photocatalytic treatment as indicated by presence of peaks at 264 and 373 nm. However, after treating with ZnO/GB and I:ZnO/GB hybrid, these peaks vanished, suggesting that Cr(VI) was reduced to Cr(III) and removed by adsorption onto the functionalized glass beads (Figure 10).

**FIGURE 11:**
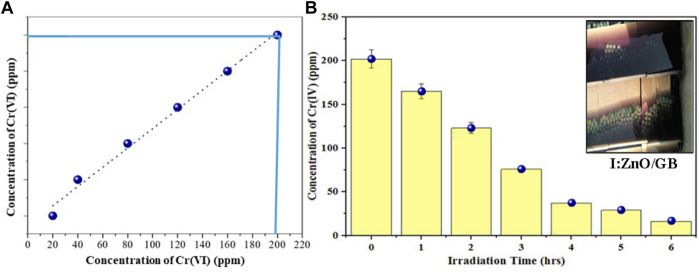
**(A)** Concentration of Cr(VI) in real tannery effluent determined by UV/visible spectroscopy. **(B)** Decrease in concentration of Cr(VI) after the solar photocatalytic reduction reaction in 6 h.

In the real tannery effluent sample, the concentration of Cr(VI) was determined to be 200 ppm by UV/vis spectroscopy (Figure 11A). Under the reaction conditions optimized by RSM, the solar photocatalytic reaction has been executed using I:ZnO/GB for 6 h under natural direct sunlight. After 5 h of contact of tannery effluent with the immobilized solar photocatalyst, a decrease in concentration of Cr(VI) was measured to be 200 ppm which reduced to 5 ppm after 6 h (Figure 11B). The real tannery effluent was blue in color due to Cr(VI) and other components, and after the photocatalytic reduction reaction under sunlight, effluent was decolorized, indicating the substantial decrease in the concentration of Cr(VI). According to previous research studies, Cr(III) is less toxic, rather a trace nutrient and desirable in water. According to USE, the Cr(VI) contaminant level is 0.1 ppm (Navin et al., 2021)

The removal of Cr from real tannery effluent was also measured by AAS. The concentration of Cr as Cr(VI) and Cr(III) was found to decrease with time. The samples collected from the reaction mixture containing I:ZnO/GB were used for determination of Cr concentration (Table 2). It can be concluded from the results obtained that with respect to time concentration of Cr decreased sharply after 3 h and became almost constant after 6 h. This can be attributed to both increases in the concentration of H^+^ with time to increase the rate of reduction of Cr(VI). Moreover, the adsorption of Cr(III) onto the surface of I:ZnO/GB after solar photocatalytic reduction decreased overall concentration of Cr in effluent.

**TABLE 2:** Concentration of Cr in ppm, determined by AAS with respect to time of the solar photocatalytic reaction.

Sample collection time	0 min	60 min	120 min	180 min	240 min	300 min
Concentration of Cr in ppm	200 (ppm)	160 (ppm)	120 (ppm)	60 (ppm)	20 (ppm)	5 (ppm)

### Mechanism of Cr(VI) to Cr(III) Reduction by I:ZnO/GB Hybrid

As delineated in UV/vis spectroscopy-based results, iodine doping elevated visible light harvesting potential of ZnO nanodiscs and accelerated the visible light-activated photocatalytic reduction process. This achievement can be attributed to induction of high density of defect levels in the bandgap region, causing efficient separation of charge carriers and remarkable reduction in time span of their interfacial transfer (Barka-Bouaifel et al., 2011; Umar et al., 2019).

The possible mechanism of Cr(VI) reduction by I:ZnO has been also mentioned in the Eqs. 2–4. After adsorption in dark, the aqueous sample of Cr(VI) was brought in contact with pure ZnO and I:ZnO and irradiated with natural sunlight for photocatalytic reduction. In the presence of sunlight, electron hole pairs were generated first (Eq. 2). In the next step, hole reacted with the water molecule to generate extremely reactive H^+^ (Eq. 3). Finally, these reactive H^+^ and electrons collectively reduced Cr(VI) to Cr(III) (Eq. 4). The overall mechanism showed that the surface area of I:ZnO played a vital role in the photocatalytic reduction of Cr(VI) to Cr(III) under solar irradiation.

$$I:ZnO+Irradiation\;\to \;I:ZnO\left(h_{VB}^{+}+e_{CB}^{-}\right)$$ (2)

$$H_{2}O+h^{+}\to {\;}^{\cdot }OH+H^{+}$$ (3)

$$CrO_{4}^{2-}+8H^{+}+\;3e^{-}\to \;Cr^{3+}+4H_{2}O$$ (4)

Parallel results were also reported by different researchers (Bhati et al., 2019; Islam et al., 2019). I:ZnO proved to be an efficient catalyst under the existing situation of environmental pollution with heavy metal ions and other toxic pollutants (Sasmaz et al., 2016; Sasmaz and Sasmaz, 2017; Yildirim and Sasmaz, 2017; Igwe and Nwamezie, 2018; Palutoglu et al., 2018; Sasmaz et al., 2018; Deeba et al., 2019; Iwuoha and Akinseye, 2019; Adetutu et al., 2020; Awwad et al., 2020). There is a dire need to develop and utilize an efficient photocatalyst which is active under solar light irradiation since it is cost-free to be used for effluent treatment (Ashar et al., 2021).

Photocatalytic reduction of Cr(VI) to Cr(III) by I:ZnO/GB hybrid was compared with undoped and doped ZnO that were most commonly employed for the reduction of Cr(VI) in effluent (Table 3). From the comparison in results, it is quite obvious that I:ZnO/GB hybrid possesses 10–50% higher photocatalytic efficiency than composites of ZnO reported in the literature, suggesting I:ZnO/GB hybrid as a potential candidate to be applied for the photocatalytic reduction of toxic Cr(VI) to Cr(III).

**TABLE 3:** Comparative analysis of photoreduction of Cr(VI) to Cr(III) by different photocatalysts with I:ZnO/GB hybrid.

Sr No	Photocatalyst used	Metal concentration removed (ppm)	Source of irradiation	% removal	Contact time (min)	Reference
1.	I:ZnO/GB hybrid	30	Sunlight	98	240	**Our study**
2.	ZnO/kaolin composite	30	UV light	∼80	120	Shirzad-Siboni et al. (2014)
3.	ZnO	30	UV light	∼36.7	180	Joshi and Shrivastava, (2011)
4.	ZnO	50	Visible light	∼40	60	Le et al. (2019)
5.	Ag/ZnO	50	UV light	∼68	60	sang-aroon et al. (2014)
6.	ZnO	20	UV light	∼60	120	Shirzad Siboni et al. (2011)
7.	Bi/ZnO nanocomposites	50	Visible light	∼75.5	180	Yuan et al. (2018)

### Reusability of I:ZnO/GB as a Solar Photocatalyst

**FIGURE 12:**
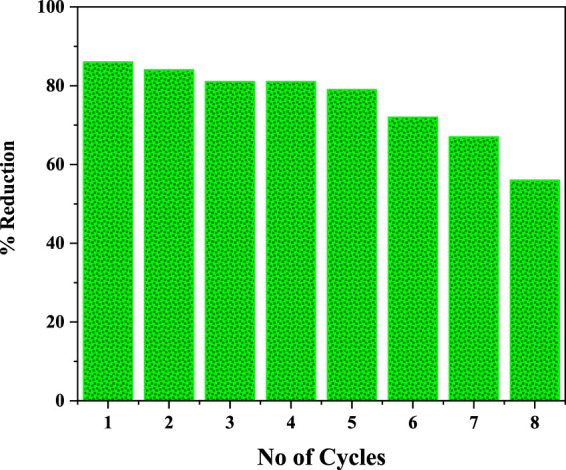
Cycles of the solar photocatalytic reaction for % reduction of Cr(VI) to determine reusability of I:ZnO/GB.

The solar photocatalytic reaction efficiency of I:ZnO/GB was investigated for eight cycles, and reduction % of Cr(VI) to Cr(III) was compared. It was found that the solar photocatalyst retained its efficiency for five cycles in context to % reduction of Cr(VI) in real tannery effluent. After that, a slight decrease in % reduction was observed, and it kept on decreasing gradually for seventh and eighth cycles. It can be concluded that grafting of glass beads with I:ZnO has been found durable and well adhered. During the photocatalytic reaction, erosion of the photocatalyst from the etched surface was observed after six cycles but was found negligible. This can be attributed to the method of grafting adopted (Figure 12).

## Conclusion

Intrinsic ZnO and I:ZnO were grafted onto the surface of etched glass beads through the SILAR method as ZnO/GB and I:ZnO/GB. The XRD pattern and micrographs showed a crystalline hexagonal wurtzite, monocrystalline structure of ZnO nanodiscs and I:ZnO nano-bricks. The optical properties of fabricated samples exhibited higher sunlight harvesting capability of I:ZnO than intrinsic ZnO. As determined by inserting DRS results in the Kubelka–Munk equation, the bandgap energy of I:ZnO decreased to 2.9 eV. The thermal stability of ZnO and I:ZnO compared by TGA delineated 14.7% higher thermal stability of the iodine-doped sample of ZnO. The photocatalytic efficiency of ZnO and I:ZnO/GB hybrid was evaluated for the reduction of Cr(VI) in standard solutions and tannery effluent under sunlight irradiation. Photocatalytic reduction efficiency of Cr(VI) to Cr(III) was investigated to be enhanced on the employment of I:ZnO nano-bricks as compared to intrinsic ZnO nanodiscs. According to the response of RSM runs, the pH change showed that photocatalytic reduction efficiency of Cr(VI) in acidic to neutral pH was much greater than alkaline pH. Moreover, on the increase in initial concentration of Cr(VI) up to 30 ppm and sunlight irradiation time of 3 h, the rate of reduction was enhanced. However, further increase in concentration of Cr(VI) led to the decrease in the reduction efficiency of I:ZnO/GB. Complexation of Cr(VI) and of Cr(III) with 1,5-DPC also confirmed the remarkable reduction of Cr(VI). The decrease in concentration of Cr in real tannery effluent has further been examined under optimized reaction conditions by UV/vis spectroscopy and AAS. The results of solar photocatalytic reduction before and after treatment of effluent have been reported with respect to time using I:ZnO/GB. Reusability of I:ZnO/GB proved it as a promising solar photocatalyst which can be used efficiently and economically for reduction of Cr(VI) in tannery effluent under natural sunlight.

## Data Availability

The raw data supporting the conclusion of this article will be made available by the authors, without undue reservation.
